# Prevalence and factors associated with exclusive breastfeeding among rural mothers of infants less than six months of age in Southern Nations, Nationalities, Peoples (SNNP) and Tigray regions, Ethiopia: a cross-sectional study

**DOI:** 10.1186/s13006-020-00267-y

**Published:** 2020-04-10

**Authors:** Dawit Hagos, Amare Worku Tadesse

**Affiliations:** 1 Save the Children South Sudan, Juba, South Sudan; 2 Department of Reproductive Health, Population and Nutrition, Addis Continental Institute of Public Health, Addis Ababa, Ethiopia; 3 Department of Global Health and Population, Harvard T.H. Chan School of Public Health, Boston, MA USA

**Keywords:** Exclusive breastfeeding, Rural, SNNPR, Tigray, Ethiopia

## Abstract

**Background:**

Exclusive breastfeeding (EBF) is the global recommended nutrition for infants less than 6 months of age. The prevalence of exclusive breastfeeding in Ethiopia is much lower than the recommendations of World Health Organization (WHO). This study aimed to assess the prevalence and associated factors of EBF among rural mothers of infants less than 6 months of age in two regions, Southern Nations, Nationalities and Peoples (SNNP) and Tigray Regions, of Ethiopia.

**Methods:**

The research was based on the secondary data analysis of community-based cross-sectional study conducted in 56 rural woredas (districts) in two regions of Ethiopia for impact evaluation of Alive & Thrive multi-year project. The 24-h recall dietary data were collected from 600 mother-infant dyads where the infant was less than 6 month of age, using multistage cluster sampling technique and 584 dyads were found eligible for analysis. Bivariate and multivariable logistic regressions were applied to identify the associated factors of exclusive breastfeeding.

**Results:**

The prevalence of EBF of infants less than 6 months of age was 88.0% (95% CI 84.9, 90.4%). The odds of practicing EBF were significantly higher among infants less than 2 months of age (AOR 4.47, 95% CI 2.41, 8.27), married mothers (AOR 4.35, 95% CI 1.50, 12.67), mothers who gave birth in health facilities (AOR 2.07, 95% CI 1.15, 3.73) and mothers who received breastfeeding counseling during pregnancy (AOR 2.23, 95% CI 1.26, 3.96).

**Conclusions:**

The prevalence of EBF was relatively high when compared with previous studies in Ethiopia but close to the WHO recommendations of 90 %. Infant age, marital status of mothers, delivery place and breastfeeding counseling during pregnancy were identified as factors associated with EBF practices in 24 h preceding the survey. Devising appropriate strategies on breastfeeding messaging/counseling and support in addressing poor breastfeeding practices through existing government-led health intervention packages are recommended.

## Background

Global recommendations for optimal infant feeding include exclusively breastfeeding (EBF) for the first 6 months of their life [1–4]. Research findings have shown up to a 13% reduction in infant mortality rate can be achieved through the practices of EBF in low income countries, including Bangladesh and Pakistan [5], in addition to infant and maternal health benefits [2, 6, 7].

Despite the well recognized multiple benefits of exclusive breastfeeding, the coverage of exclusive breastfeeding is very low globally as compared to the WHO recommendation of 90% coverage. Trend data analysis on prevalence of exclusive breastfeeding among infants less than 6 months of age in developing countries indicated that only 39% of the infants were exclusively breastfed in 2010 [8]. The same study has estimated the prevalence of EBF in Africa and Asia as 35 and 41% [8].

In Ethiopia, breastfeeding is almost universal practice among mothers but the proportion of exclusively breastfed infants less than 6 months of age was low at 58% in 2016 [9]. Hence, the nation may benefit less from the multiple advantages of exclusive breastfeeding for infants, mothers and the nation in general.

Exclusive breastfeeding of infants less than 6 months of age is associated with many individual, group and societal level factors that can be further classified as infant, maternal and household characteristics, and health service-related factors [10–13]. In Ethiopia, infant characteristics including age and prelacteal feeds; maternal characteristics such as age, education, occupation, and marital status; and health facility characteristics including place of delivery, antenatal and postnatal care and counselling were previously reported to influence EBF [14–18]. Strategies to promote exclusive breastfeeding are desired at the national, health facility and community levels [4].

Therefore, this study aimed to assess the prevalence of exclusive breastfeeding among infants less than 6 months of age in the past 24 h preceding the survey date and to determine the factors associated with EBF in two regions of Ethiopia, Southern Nations, Nationalities and Peoples (SNNP) and Tigray regions.

## Methods

This study was based on analysis of the secondary data from the impact evaluation of Alive & Thrive project implemented in Ethiopia in collaboration with Ethiopian Ministry of Health, Integrated Family Health Program (IFHP) and other local organizations. This multi-year project (2009–2014) was implemented in 89 woredas (local administrative units similar to districts) of the two regions of Ethiopia: SNNPR (54 woredas) and Tigray Region (35 woredas). Baseline, mid-term evaluation and impact evaluation studies were conducted based on the project time frame.

As part of the impact evaluation, the data were collected in SNNP and Tigray regions within the frame work of an impact evaluation with adequacy design that captures changes overtime in the absence of a control group. Hence, community-based cross-sectional study design was deployed using multistage cluster sampling techniques to generate the primary data. A total of 75 enumeration areas (EA) were selected for the final evaluation of the project from 56 target woredas that included 37 woredas from SNNPR and 19 woredas from Tigray Region. The study population was infants less than 6 months of age, living in 75 enumeration areas of the 56 woredas and data were collected from 600 sample infant mother dyads. All data were obtained from face to face home interview with mothers of infants less than 6 months of age and 584 of them found eligible for analysis. The incomplete data of 16 infant mother dyads were excluded from analysis due to missing data on important variables of the study.

The sample size required for this study was calculated using the formula to estimate single population proportion for objective 1 based on the EDHS 2011 finding of 52% prevalence of exclusive breastfeeding for 6 months [19] with an estimated missing data of 10% that resulted sample size of 427.

The sample sizes for the second objective was calculated based on previous findings of prevalence of prelacteal feeding of 19.9% [14], prevalence of initiation of breastfeeding within the first 1 h of birth (72.3%) and mothers receiving advice about EBF (90.2%) [10]. The sample size calculated for EBF counselling during antenatal care (ANC), delivery and postnatal care (PNC) (520 infants less than 6 months of age) was the highest sample size and taken as the sample size for both objective 1 and 2. However, all the 600 data sets of mothers who had infants less than 6 months of age in the end line evaluation of 2014 were considered in this analysis. The investigator checked all the data for completeness and accuracy. Data set of 584 infants was found eligible for analysis after data cleaning. The 24-h dietary recall method was used to measure exclusive breastfeeding.

### Variables

The dependent variable for this study was exclusive breastfeeding of infants less than 6 months of age based on the WHO definition as the proportion of infants less than 6 months of age who are fed with breast milk exclusively without any other drinks or foods in the past 24 h with the exception of oral rehydration salt (ORS), vitamin and mineral supplements, or medicine [20].

A wide range of independent variables were assessed as potential factors that influence exclusive breastfeeding. Based on conceptual framework described by Hector [13], we studied age and sex of infant, maternal age, education, marital status, occupation, knowledge of mothers in EBF, infant feeding practices and birth experience. In addition, health facility services variables including ANC and PNC, breastfeeding counseling in the health facilities, place of delivery, type of delivery and breastfeeding support were studied.

### Statistical analysis

Data were analysed using SPSS 20 for Windows statistical software package (International Business Machines Corporation, New York, NY, USA). Descriptive statistics were applied for sociodemographic variables describing the characteristics of infants, mothers and the households, and to determine the prevalence of exclusive breastfeeding practices, timely initiation of breastfeeding and prelacteal feeding. Proportions were compared by exclusive breastfeeding using correlation of independent variables. To determine the association between independent variables and the dependent variable (EBF), bivariate logistic regression was carried out for selected variables with the outcome variable (EBF). Hosmer and Lemeshow test for goodness of fit was conducted and the model was found fit with *P -* value > 0.05 (chi-square 6.266, *p* = 0.618). Variables with a *p* - value less than 0.2 on bivariate logistics regression analysis were included in the multivariable logistic regression to determine associated factors with EBF. The statistical significance was determined using odds ratio of 95% confidence interval with *p* - value less than 0.05.

## Results

### Sociodemographic characteristics

Data of 584 mothers having children less than 6 months old were analyzed and the sociodemographic characteristics were depicted in Table 1. About one third (32%) of the mothers were young (15–24 years) and the median age of the mothers was 27 years (mean 27.8 + 6.4 years). Majority (95.9%) of the mothers were married. More than half (54%) of the mothers had completed primary school or above. Thirty-seven percent of the infants were two to 3 months old and the median age was 2 months (Table 1).

**Table 1:** Sociodemographic characteristics of mothers of infants less than 6 months of age, Tigray & SNNPR, (*n* = 584)

Variables	Numbers (***n***)	Percent (%)
Age of mothers in years		
15–24	186	32.0
25–29	158	27.2
30–34	124	21.3
35–49	113	19.5
Age of child in months		
0–1	191	32.7
2–3	217	37.2
4–5	176	30.1
Sex of child		
Male	286	49
Female	298	51
Religion of Mother		
Orthodox Christian	264	45.2
Protestant	285	48.8
Muslim	27	4.6
Others	8	1.4
Mothers educational status		
Never attend school	262	46.0
Primary and above	308	54.0
Marital status		
Single	10	1.7
Married	560	95.9
Divorced/separated	14	2.4
Mothers occupation		
Farmer	30	5.2
Employed	9	1.5
Self-employed	36	6.2
House wife	498	85.4
Others	10	1.7

### Health service characteristics

As indicated in Table 2, most (90.4%) of the mothers attended antenatal care during their last pregnancy and more than half (50.7%) of them had four or more visits. However, only 58.4% of the mothers received breastfeeding counseling during their pregnancy of the index child. About 55 % (54.7%) of the mothers delivered in the health facility while the rest of the mothers gave birth at home. The majority (97.8%) of the mothers had a vaginal birth. Only 35.3% of the mothers had got support from anyone (health workers, health extension workers, volunteers, family members, neighbors and friends) within the first 1 h of birth. Three quarters (76.1%, *n* = 190/249) of these mothers have got the support in the nearby health facilities from health staff or health extension workers within the first 1 h of birth or more.

**Table 2:** Infant feeding and health service characteristics among mothers of infants less than 6 months of age, Tigray & SNNPR, (*n* = 584)

Variables	Numbers (***n***)	Percent (%)
ANC follow up		
Yes	527	90.4
Frequency of ANC visits		
Once	38	7.3
Twice	81	15.6
3 times	137	26.4
> 4 times	263	50.7
Breastfeeding counseling during pregnancy		
Yes	340	58.4
Place of Delivery		
Home	263	45.3
Health facility	318	54.7
Mode of delivery		
Vaginal delivery	571	97.8
Cesarean section	13	2.2
Did anyone help you with breastfeeding?		
Yes	251	43.1
Timing of support on breastfeeding received		
No support	333	57.0
Within 1 h	206	35.3
Within 24 h	35	6.0
After 24 h	10	1.7
Where did you get the support?		
Home	59	23.7
Health facility	190	76.3
Initiation of breastfeeding		
Within 1 h	479	82.6
Above 1 h	101	17.4
Prelacteal feeding		
Yes	9	1.5
Frequency of breastfeeding		
< 8 times per day	41	7.1
> 8 times per day	536	92.9
Colostrum feeding		
Yes	515	88.3
Is the child still breastfeeding?		
Yes	580	99.5
Breastfeeding problem		
Yes	20	3.4
Mother’s knowledge of breastfeeding initiation		
Within 1 h	515	88.6
Within 24 h	66	11.4
Mother’s knowledge about colostrum feeding		
Discarding	120	20.6
Feeding to the new baby	462	79.4
Exclusive breastfeeding practices		
Yes	513	88.0

### Breastfeeding practices

**Fig. 1:**
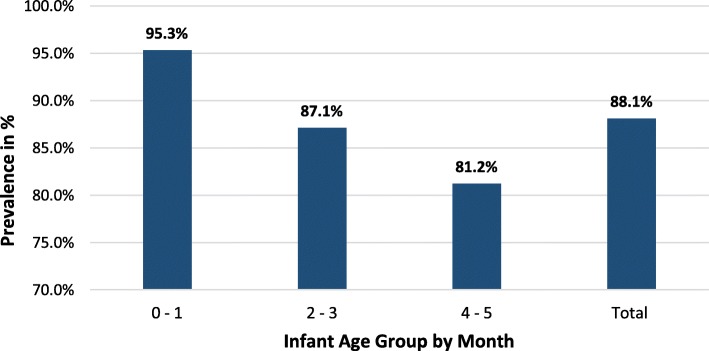
Prevalence of exclusive breastfeeding by age group of infants less than 6 months of age, SNNPR & Tigray Region, (*n* = 584)

Almost all (99.5%) of the infants were still breastfeeding during the survey period but only 88.0% of the mothers exclusively breastfed in the past 24 h of the survey date (Table 2). The prevalence of exclusive breastfeeding was relatively high among infants 0–1 months old (95.3%) (Fig. 1). Most of the mothers (82.6%) initiated breastfeeding within the first 1 h of birth and 1.5% mothers gave prelacteal foods (plain water, sugar/glucose water, raw butter and infant formula) within the first few hours of birth (Table 2). The majority (88.3%) of the mothers gave the first breast milk (Colostrum) to their young babies while the rest 11.7% were discarded it. Ninety-three percent of the mothers breastfed the child more than eight times per day.

Mother’s awareness on timely initiation of breastfeeding and colostrum feeding were assessed and 88.6% of the mothers knew that breastfeeding should be initiated within the first 1 hour of birth (Table 2). More than three quarters (79.4%) of the mothers believed that colostrum should be given to the new baby while the rest 20.6% of the mothers believed that it has to be discarded before the child starts breastfeeding (Table 2).

Bivariate and multivariable analysis of sociodemographic, infant feeding practices and health services related factors affecting exclusive breastfeeding were carried out. Age of the infant, marital status of mothers, breastfeeding counseling during pregnancy and place of delivery have showed an association with practices of exclusive breastfeeding in the past 24 h. The multivariate analysis revealed that infants less than 2 months of age were four times (AOR = 4.47, 95% CI 2.41, 8.27) more likely to be exclusively breastfed than 3–5 months old. The likelihood of practices of exclusive breastfeeding was four times (AOR = 4.35, 95% CI 1.50, 12.67) higher among married women than the unmarried. The odds of practice of exclusive breastfeeding was double among mothers who gave birth in health facilities (AOR = 2.07, 95% CI 1.15, 3.73) and received breastfeeding counseling during pregnancy (AOR = 2.23, 95% CI 1.26, 3.96) as compared to their counterparts (Table 3).

**Table 3:** Association of sociodemographic, infant feeding practices and health services related characteristics with EBF of infants less than 6 months of age, SNNPR & Tigray Region, (*n* = 572)

Variables	Exclusive Breastfeeding		COR CI (95%)	AOR CI (95%)
	No (n, %)	Yes (n, %)		
Age of mothers in years				
15–29	34 (9.9%)	309 (90.1%)	1.75 (0.94, 3.25)	1.69 (0.97, 2.95)
30–49	35 (14.8%)	202 (85.2%)	1.00	1.00
Age of child in months				
0–2	17 (5.6%)	288 (94.4%)	4.39 (2.34, 8.24) ^***^	4.47 (2.41, 8.27)^***^
3–5	53 (19.1%)	225 (80.9%)	1.00	1.00
Sex of child				
Male	38 (13.3%)	247 (86.7%)	0.96 (0.55, 1.69)	–
Female	32 (10.7%)	266 (89.3%)	1.00	
Mothers’ educational status				
Never attend school	34 (13.0%)	228 (87.0%)	1.00	–
Primary and above	32 (10.4%)	275 (89.6%)	0.97 (0.53, 1.76)	
Mothers’ occupation				
Working mothers	11 (13.1%)	73 (86.9%)	1.00	
House wife	58 (11.6%)	440 (88.4%)	1.09 (0.50, 2.39)	–
Marital status				
Unmarried	6 (26.1%)	17 (73.9%)	1.00	1.00
Married	64 (11.4%)	496 (88.6%)	5.25 (1.64, 6.79)^**^	4.35 (1.50, 12.67)^**^
ANC follow up				
No	6 (10.7%)	50 (89.3%)	1.00	1.00
Yes	63 (12.0%)	463 (88.0%)	0.48 (0.17, 1.30)	0.43 (0.16, 1.14)
Breastfeeding counseling during Pregnancy				
No	37 (15.4%)	204 (84.6%)	1.00	1.00
Yes	32 (9.4%)	308 (90.6%)	2.16 (1.16, 4.02)^*^	2.23 (1.26, 3.96)^**^
Place of delivery				
Home	41 (15.6%)	222 (84.4%)	1.00	1.00
Health facility	28 (8.8%)	289 (91.2%)	2.20 (1.13, 3.29)*	2.07 (1.15, 3.73)*
Mode of delivery				
Vaginal delivery	67 (11.8%)	503 (88.2%)	3.21 (0.57, 17.97)	3.22 (0.60, 17.27)
Cesarean section	3 (23.1%)	10 (76.9%)	1.00	1.00
Initiation of breastfeeding				
Within 1 h	50 (10.4%)	429 (89.6%)	1.66 (0.84, 3.27)	1.72 (0.90, 3.29)
Above 1 h	18 (18.0%)	82 (82.0%)	1.00	1.00
Prelacteal feeding				
No	66 (11.5%)	506 (88.5%)	4.08 (0.89, 18.68)	4.33 (0.98, 19.09)
Yes	3 (33.3%)	6 (66.7%)	1.00	1.00
Colostrum feeding				
No	12 (17.6%)	56 (82.4%)	1.00	
Yes	57 (11.1%)	457 (88.9%)	1.32 (0.58,2.99)	–
Breastfeeding problem				
No	67 (11.9%)	495 (88.1%)	1.20 (0.24,6.13)	–
Yes	3 (15.0%)	17 (85.0%)	1.00	
Did anyone help you with breastfeeding?				
No	43 (13.0%)	288 (87.0%)	1.00	
Yes	26 (10.4%)	224 (89.6%)	0.84 (0.43, 1.63)	–

* P < 0.05, ** P < 0.01, *** P < 0.001

*COR* Crude Odds Ratio, *AOR* Adjusted Odds Ratio, *CI* Confidence Interval

## Discussion

The results of the study showed a prevalence of EBF of 88.0% (95% CI 84.9–90.4%) which was close to the WHO recommendations of 90%. Age of the infant, marital status of the mothers, breastfeeding counseling during pregnancy and place of delivery were identified as significantly associated factors of exclusive breastfeeding practice among mothers of infants less than 6 months of age in the past 24 h preceding the survey date.

The prevalence of EBF among rural mothers in SNNPR and Tigray region was much higher than the national average of EDHS 2011 (52%), the targets of HSDP IV of the Federal Ministry of Health of Ethiopia (70%) for 2015 [19], average estimates for Eastern and Southern African region (47%) and developing countries (39%) [8]. It was also much higher than cross-sectional studies conducted in some parts of Ethiopia [14–17]. This might attribute to the variations in breastfeeding promotion interventions of the health extension system with the support of the Integrated Family Health Program (IFHP) of Alive & Thrive and others. The current finding was also higher than EBF in Nigeria [10, 21], rural Egypt [22], Uganda [11], East and South East Asia countries [23]. The variation can be explained by time of the study, study design, variations in dietary/EBF assessment method, age distribution of infants included in the study, sociodemographic, economic and cultural differences across the study areas.

In this study, infant age, delivery place, marital status of the mother and breastfeeding counseling during pregnancy were identified as associated factors with EBF in rural areas of SNNP and Tigray regions. Previous studies conducted in Ethiopia and other countries indicated that sex of the infant [21, 22], age of the mother [15, 22, 24], maternal education [10, 18, 24] occupation of the mother [14–17], ANC [10, 14] and PNC [14, 15] practices of the mother were reported as predictors of EBF among infants less than 6 months old. However, none of these factors were associated with EBF in this study which could be due to the socio-cultural differences on breastfeeding among the study participants, study design or the sample size might not be able to capture the association between the aforementioned factors and exclusive breastfeeding.

Infants less than 3 months of age have higher chance of EBF (AOR 4.47, 95% CI 2.41, 8.27) than the older infants. Research findings from Nigeria [7, 10, 21], Canada [24] and Ethiopia [15, 16, 18] were also in line with this study confirming that EBF decreases significantly as age of the infant increases. This can be explained by the traditional postpartum care provided to the mother within the few months after birth which gives higher contact with the infant to breastfeed, as the mothers stay at home and are less engaged in the household chores.

Married mothers practiced EBF four times (AOR 4.35, 95% CI 1.50, 12.67) higher than unmarried mothers. Evidences from Tanzania [25], Norway [26] and Canada [24] supported the current finding. The possible reason could be married mothers get support to practice EBF from their partners and other family members. In contrary, previous study conducted in Ethiopia has shown unmarried mothers practiced significantly higher EBF (AOR 2.3, 95% CI 1.1, 4.7) than married mothers [18]. This might be due to differences in study area, design and sample size.

Mothers who gave birth in health facilities practiced EBF twice more (AOR 2.07, 95% CI 1.15, 3.73) than the mothers who delivered at home. This result was supported by reports from Uganda [11], Ghana [12] and Ethiopia [15, 17]. This could be explained as mothers who give birth in health facilities have more opportunities to obtain breastfeeding support including counselling on the benefits of breastfeeding, correct positioning and attachment. The mothers could have a better chance to get better information of exclusive breastfeeding from the health facilities because of the availability of IEC/BCC (Information Education Communication or Behavior Change Communication) materials in the facilities. However, a national study conducted in Canada differed with the findings of the current study because mothers who preferred to deliver at home were more likely to practice EBF (AOR 5.29, 95% CI 2.95, 9.46) [24]. This could be due to the differences of the health services delivered at various settings.

Mothers who received breastfeeding counseling during pregnancy practiced EBF double (AOR 2.23, 95% CI 1.26, 3.96) of their counterparts which is consistent with reports from Ethiopia [14, 15, 17] and Uganda [11]. The possible reason could be the information provided during counseling may help the mothers to increase their awareness about the importance of breastfeeding that brought attitudinal changes on exclusive breastfeeding. The expansion of health facilities in rural areas with trained health professionals, and the involvement of the Women Development Army (WDA) in the health extension intervention, who transmit messages of appropriate breastfeeding to the mothers could contribute a lot.

### Limitations of the study

This study shares the limitation of any other cross-sectional studies of drawing cause and effect relationship between the factors and the prevalence of exclusive breastfeeding in the study area. It was not possible to analyze the prevalence of EBF for the two regions separately as the sample size were calculated for the combined regions as the domain of IFHP project area. The prevalence of EBF might be overestimated due to the nature of the method used for data collection: the 24-h recall method. Some of the independent variables were not associated with the dependent variable which has been reported as factors associated with exclusive breastfeeding in many studies. This might be due to the sample size which was not able to justify the relationship between the factors and the dependent variable. This study is representative of rural parts of the two regions; hence it cannot be generalized to the entire population of the two regions. Therefore, the interpretation of the study should consider the strengths and the limitations of the study.

## Conclusions

The prevalence of exclusive breastfeeding among mother of infants less than 6 months of age in the two regions was relatively high as compared with previous studies but was in line with the WHO recommendations. In this study, exclusive breastfeeding practices of mothers in the past 24 h were affected by infant age, marital status of mothers, place of delivery and breastfeeding counseling during pregnancy. Devising appropriate Social and Behavior Change Communication (SBCC) strategies on breastfeeding messaging/counseling and support in addressing poor breastfeeding practices through the existing government-led health intervention packages such as the community-based health extension program are recommended. Breastfeeding messaging during antenatal care and postnatal care, breastfeeding support during pregnancy, and establishment of mother to mother support groups could be the potential areas for improvement.

## Data Availability

The data used and/ or analysed for the current study are available from the second author upon reasonable request Please contact Dr. Amare Worku to request the data at adtadesse@gmail.com.
